# Association Between Insurer Connectivity in Appalachian Population Health Networks and Preventable Hospitalizations: Evidence from Kentucky

**DOI:** 10.13023/jah.0502.03

**Published:** 2023-08-01

**Authors:** Rachel Hogg-Graham, Kelsey R. Gatton, Rick Ingram, Glen P. Mays

**Affiliations:** University of Kentucky, College of Public Health, rachel.hogg@uky.edu; University of Kentucky, College of Public Health; University of Kentucky; Department of Health Systems, Management and Policy, Colorado School of Public Health

**Keywords:** Appalachia, population health systems, preventable hospitalizations, rural health

## Abstract

**Introduction:**

Addressing complex health and social needs requires cross-sector collaboration to deliver medical, social, and population health services at the community level. Capacity in community health and social services networks may be constrained in regions like Appalachia due to the combined effects of rurality and persistently poor health and social outcomes. One way that cross-sector networks serving low-resource communities can expand their capacity is by engaging partners, like health insurers, who can leverage resources from outside the local area.

**Purpose:**

This study examines insurer connectivity in cross-sector networks across Kentucky’s geographic regions and the association between connectivity and the probability of an individual experiencing a preventable hospitalization.

**Methods:**

A cross-sectional design was used that linked data from the National Longitudinal Survey of Public Health Systems (NALSYS) with 2018 patient-level Kentucky hospital discharge data to examine the association between insurer connectivity in community networks and preventable hospitalizations across urban, rural non-Appalachian, and Appalachian regions.

**Results:**

Analysis of the data shows substantial geographic variation in the association between insurer connectivity in community networks and preventable hospitalization. Insurer connectivity in rural Appalachian communities was associated with lower likelihood that an individual was admitted for a preventable hospitalization ( *p* < 0.01).

**Implications:**

Findings suggest insurer connectivity in cross-sector community health and social services networks has the potential to strengthen network capacity to address preventable hospitalizations and improve health outcomes and well-being for the people of Appalachia.

## INTRODUCTION

Social determinants of health—such as food insecurity, housing instability, and household financial strain—contribute to high levels of disease burden and premature mortality in the U.S., but medical providers often lack the resources and expertise needed to address these issues effectively.^1^ Addressing complex health and social needs requires cross-sector collaboration to deliver medical, social, and population health services at the community level. Cross-sector partnerships can help ensure that patients receive assistance from the providers best equipped to deliver the needed services and supports, whether that is clinical care from a local physician or food assistance from a community-based nonprofit.^2^,^3^ The ability to successfully move patients through community health and social services networks hinges on both links between organizations and sufficient community capacity across all sectors.^4^

Rural communities are likely to face limited capacity in community health and social services networks due to numerous unique characteristics. Rural areas have fewer healthcare providers, less access to quality clinical care, and limited social resources, such as housing, food and transportation assistance programs.^5^ Recent research has also found growing disparities between rural and urban communities in the delivery of population health activities by public health agencies and their networks of community partners.^6^ These activities include community-wide initiatives to assess health needs and risks, develop health improvement priorities and plans, and connect community residents to needed health and social services. Capacity in community health and social services networks may be particularly constrained in regions like Appalachia due to the combined effects of rurality, persistent economic deprivation, population loss, and elevated health and social needs.^7^,^8^

One way that cross-sector networks serving resource-constrained communities can expand their capacity is by engaging partners who can leverage resources from outside the local area. Health insurers, for example, typically serve large geographic service areas within and across states and have access to expertise, information, and financial assets that often exceed the resources available to more localized community-based organizations. Insurers may contribute to the community networks operating within their service areas to improve the health and social resources available to their members, possibly making larger contributions to networks and communities with greater unmet needs. This overlooked form of cross-subsidization may play a role in strengthening networks in areas with less community health and social services network capacity.^9^,^10^ Insurers can bring unique resources and data to community-level efforts seeking to integrate health and social services.^2^ For example, utilization data can be used to pinpoint hotspots of high cost and use among enrollees. Increasingly, insurers are also engaging in the screening of enrollees for unmet social needs, providing the opportunity to examine both care and social needs patterns across enrollee populations.^1^,^11^ These data may be particularly powerful in regions like Appalachia given the long-standing disparities in access to care and outcomes when compared to other regions in Kentucky. Increasing insurer involvement in community health and social services networks may be one strategy to improve both health outcomes and community capacity.

Evidence on the association between insurer participation in community health and social services networks and improved health outcomes is limited. To help fill this gap, this study used hospital discharge data linked with health and social services network data to examine the association between insurer connectivity in cross-sector networks and preventable hospitalizations across geographic regions in Kentucky. Kentucky was selected for this analysis based on both the availability of comprehensive community network data and the geographic diversity of the state. It has three distinct urban, rural non-Appalachian, and rural Appalachian regions. Preventable hospitalizations was selected as the primary outcome based on prior research linking hospitalizations to both patient and community-level socioeconomic conditions.^12^,^13^ Recent work by McCullough et al. also found an association between increased public health and social services spending and reductions in community-level preventable hospitalization rates.^14^ This suggests strengthening the delivery of health and social services in a community may lead to improved social needs and fewer preventable hospitalizations. We hypothesize that increased insurer engagement in community health networks will be associated with fewer preventable hospitalizations, and that association may be stronger in rural Appalachian communities.

## METHODS

Data from the National Longitudinal Survey of Public Health Systems (NALSYS) were linked with 2018 patient-level Kentucky hospital discharge data to examine the association between insurer connectivity in community health / social services networks and preventable hospitalizations across urban, rural non-Appalachian, and Appalachian regions. NALSYS is the only national and longitudinal data source that captures data on cross-sector collaboration in the delivery of population health activities.^6^,^15^–^19^ Using a validated questionnaire, NALSYS asks local public health officials to provide information about the availability of 20 core population health activities within their community and the range of sectors that deliver each activity, including hospitals, primary care providers, insurers, employers, schools, and community-based organizations. Activities in NALSYS represent a variety of nationally recommended community-level population health protections.^19^ Activities align with the core functions of public health and range from the surveillance of community health needs to the setting of community of health priorities and the associated resource allocation plans.

NALSYS data were first collected in 1998 with subsequent waves in 2006, 2012, 2014, 2016, 2018, and 2020.^19^ The original cohort of communities included a nationally representative sample with populations of 100,000 or more. The sample was expanded in 2014 to include communities serving smaller populations, as well. A statewide sample of Kentucky local public health jurisdictions (n = 61) was captured in the 2018 NALSYS data, providing comprehensive data on community health and social services networks in the state. Community networks were classified as urban (n = 20), rural non- Appalachian (n=15), and rural Appalachian (n = 26) in alignment with a previous study examining Kentucky’s networks.^8^

Using the Agency for Healthcare Research and Quality’s (AHRQ) Prevention Quality Indicators definitions, patient-level preventable hospitalization was identified via discharge data in four categories for each Kentucky county.^20^ The measures capture individuals admitted for any preventable hospitalization and three subsets of acute, chronic, and diabetes-related hospitalizations. Following the standard methodology for examining preventable hospitalizations, patients were linked back to their county of residence. The community-level health and social services network is the unit of analysis. For half of the communities in Kentucky this is a single county; the remainder consist of multi-county jurisdictions. An aggregate preventable hospitalization rate was created for the multi-county jurisdictions by summing preventable hospitalizations across all counties and dividing by the total population in the jurisdiction. The final sample included a total of 484,450 hospitalizations.

Data on insurer connectivity in the population health network were calculated using NALSYS to generate insurer *betweenness centrality*. Betweenness centrality is a common measure used in social network analysis methods that measures the extent to which one actor connects others in the network.^21^ Organizations with high betweenness centrality are often identified as brokers in the network. Betweenness centrality is measured on a scale of 0 to 1, with 1 being the highest level of connectivity. In this study, betweenness centrality was measured by examining activities shared by insurers and two other organizations (triads) in the network. Insurer betweenness centrality indicates the proportion of triads that are insurer-enhanced, allowing for identification of the potential strength of insurers to broker stronger population-health-related engagement and relationships between other organizations in the community.

Descriptive statistics were generated to examine preventable hospitalization rates and insurer connectivity in community networks across the three geographic regions. Multivariate logistic regression models were then used to identify the association between insurer connectivity and the probability of an individual being admitted for a preventable hospitalization. The primary explanatory variable was operationalized as categorical with networks having ‘no’, ‘low’, or ‘high’ insurer connectivity. Models were run first for the pooled sample including all community networks. Separate models were then run for each geographic region to determine if there were variations in associations between the three regions. Last, a set of models for the rural Appalachian region alone were run to determine if differences in the association between insurer connectivity and the probability of preventable hospitalization exist based on the patient’s coverage type. Patients were categorized into three subgroups: Medicare, Medicaid, and privately insured. Patients who were uninsured were excluded from the analysis due to small sample size.

All models controlled for a set of community socioeconomic, demographic, and healthcare system supply characteristics pulled from the Area Health Resource File (see figure notes for the full list of variables). Patient race, age, and payer were also controlled for in all models, apart from the last analysis. Standard errors were clustered at the community network level to account for patient nesting within communities.

## RESULTS

**Figure 1:**
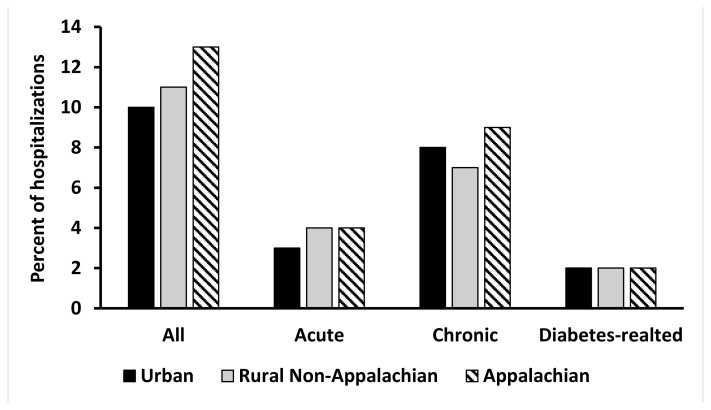
Portion of hospitalizations that were preventable by type in KY urban, non-Appalachian, and rural communities, 2018 SOURCE: Authors’ analysis of 2018 KY hospital discharge data NOTE: ‘Acute’ includes hospitalizations for community-acquired pneumonia and UTI. ‘Chronic’ includes hospitalizations for diabetes short-term complications, diabetes long-term complications, COPD or asthma in older adults, hypertension, heart failure, uncontrolled diabetes, asthma in younger adults, and lower extremity amputation among patients with diabetes. The ‘diabetes-related’ measure includes hospitalizations for diabetes short-term complications, diabetes long-term complications, uncontrolled diabetes, and lower extremity amputation among patients with diabetes. ‘All’ captures each condition listed above.

**Figure 2:**
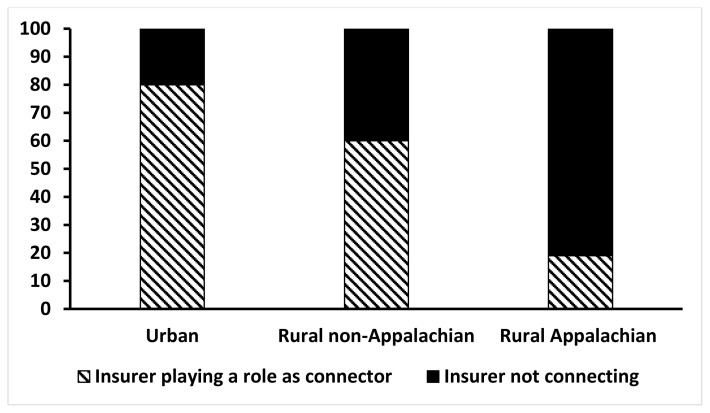
Insurer connectivity in KY community health and social services networks by geographic region, 2018 SOURCE: Authors’ analysis of 2018 NALSYS data

Preventable hospitalizations accounted for 10%–13% of hospitalizations in KY communities in 2018, with rural Appalachian communities having the highest rate (Fig. 1). Most preventable hospitalizations were for chronic conditions in all regions, with a slightly higher rate in Appalachia. Individuals were admitted at an equal rate for diabetes-related conditions in all regions. Insurers hold a position as connector in 76% of urban community health and social services networks compared to only 19% of Appalachian networks (Fig. 2). Insurers were connectors in 43% of rural non-Appalachian networks.

Results from multivariate logistic regression found statistically significant associations between high insurer connectivity and all four preventable hospitalization categories in rural Appalachian communities (Table 1). High insurer connectivity in Appalachian population health networks was associated with an almost five-percentage-point reduction in the probability of an individual experiencing a preventable hospitalization compared to those networks with no insurer connectivity (*p* < 0.01). No significant associations were found between insurer connectivity and preventable hospitalizations in urban community networks. Interestingly, low insurer connectivity in rural non-Appalachian community networks was associated with increased probability of an individual experiencing a preventable chronic hospitalization (*p* < 0.05). Otherwise, there were no statistically significant associations in rural non-Appalachian community networks.

Analysis of subgroups by payer within rural Appalachian regions found substantial variation in the association between the probability of preventable hospitalization in community networks where insurer connectivity is higher based on coverage type (Table 2, on next page). The largest associations were in Medicare and Medicaid patients, with high insurer connectivity being associated with an almost five- and six-percentage-point reduction in the probability of an individual being admitted for any preventable hospitalization (*p* < 0.01). High insurer connectivity was associated with reductions in the probability of preventable hospitalization in the acute and chronic hospitalization composites in the Medicare and Medicaid populations.

Low insurer connectivity compared to none was associated with a two-percentage- point increase in acute preventable hospitalization probability in Appalachian Medicare patients (*p* < 0.05). Similarly, high insurer connectivity was associated with an almost one-percentage-point increase in the probability of Medicare patients experiencing a diabetes-related preventable hospitalization (*p* < 0.05). Among the privately insured, high insurer connectivity was associated with reductions in the probability of all preventable hospitalization composites, apart from chronic hospitalizations. Interestingly, low insurer connectivity was associated with higher probability of experiencing a preventable hospitalization across all composites for privately insured patients in Appalachian community networks.

## DISCUSSION

Results suggest substantial geographic variation in insurer connectivity in community health / social services networks and its association with the probability of an individual being admitted for a preventable hospitalization. High insurer connectivity in rural Appalachian communities was associated with lower likelihood that an individual was admitted for a preventable hospitalization compared to those communities with no insurer connectivity. There was not a similar association in urban and rural non-Appalachian communities across the state. In fact, low insurer connectivity was associated with a higher probability of chronic preventable hospitalization in rural non-Appalachian communities.

While initially surprising, these findings may arise from several factors: rural Appalachian communities have worse health and social outcomes and may have fewer resources available to bolster capacity in community networks. High insurer connectivity in community networks may help strengthen the connections between sectors by bridging organizations that may not have previously worked together. Such action is likely to bring new resources and perspective to collaborative efforts and facilitate the meeting of both health and social needs in a complex population. Historically, more populated communities have had stronger health and social services networks that offer a greater range of services while engaging a broad set of multisector partners.^6^ It may be that collaborative efforts are more diffuse in urban areas with connectivity being driven by several partners, rather than single sectors playing a strong role. Thereby, findings do not show a statistically significant association. To provide further insight into this, betweenness centrality scores for all the sectors in NALSYS were examined, and results show that values are relatively close across all sectors in Kentucky’s urban communities. Rural non-Appalachian communities make up a small subset of networks in the state, and it may be that the sample size is not sufficient to pick up a signal.

Results from the subgroup analysis of preventable hospitalizations by payer type in rural Appalachian communities suggest that the strongest associations with insurer connectivity were concentrated in the Medicare and Medicaid patient populations. Medicare patients are at higher risk of preventable hospitalization resulting from poorly managed health conditions.^22^ Simultaneously, they are also likely to have more unmet social needs. This combination of factors makes them a high-priority population for efforts that integrate health and social services. Medicaid patients are similar in that they have complex medical and social needs. In Kentucky, a substantial portion of the population in rural Appalachian communities are enrolled in Medicaid. Although insurer headquarters are typically located in urban areas, it is possible they are willing to play the bridging role in high-need rural communities to reduce costs and improve outcomes of their enrollees. This may be part of the reason for observed instances of insurers playing a strong role in some rural Appalachian communities.

Interestingly, while there was a similar pattern in high insurer connectivity and lower preventable hospitalization probability in privately insured patients in Appalachian communities, the opposite was seen between low connectivity. It is possible that preventable hospitalization rates could be driving connectivity or vice versa—the study’s cross-sectional design does not allow for determination of directionality. If high rates of preventable hospitalizations are driving connectivity, one might expect to see reductions in preventable hospitalization over time. Future studies should take advantage of longitudinal data to provide further insight into the complexity of the relationship between cross-sector engagement and health outcomes.

A recent analysis of longitudinal trends in preventable hospitalizations rates in Kentucky suggests that the gap between rural Appalachian communities and other geographic regions is not closing, despite slight decreases in hospitalization rates.^23^ Insurer engagement in community health and social services may help to ensure these disparities do not grow, especially given the strong associations found here in Medicare and Medicaid populations. However, insurer connectivity alone may not be sufficient in closing the “Appalachian gap.” Research examining aggregate county-level preventable hospitalizations may mask some of the heterogeneity in rates based on payer type. This study’s analysis suggests strong associations between high insurer connectivity and lower probability of preventable hospitalization in Medicare and Medicaid patients. It may be that the gap between rural Appalachian and other regions is closing among these groups—and not among privately insured patients. Further research that examines these relationships among payer subgroups could highlight longitudinal variation in trends.

Several limitations should be taken into consideration alongside this analysis. The study is cross-sectional and only measures the association between insurer connectivity and the probability an individual is admitted for a preventable hospitalization. It did not explore longitudinal trends in insurer connectivity and how that might result in changes in preventable hospitalization rates. Examining engagement in community networks and health outcomes. For example, it is possible the direction and strength of relationships in communities might change over time. The study is also limited to a single state: Kentucky. Although the number of Appalachian states with available hospital discharge is limited, building a larger dataset could confirm and increase generalizability of the results.

Furthermore, the extent to which insurer market characteristics impact connectivity cannot be determined from these results. Characteristics like market concentration by payer, the number of individuals enrolled in Medicare and Medicaid, and variation in resources put forward by insures may drive connectivity. Additionally, expanding the study to include other states with rural Appalachian communities would help increase understanding of the broader relationship between insurer connectivity in community networks and health outcomes.

NALSYS collects data from the local public health official perspective, and not from that of the insurer. While prior studies have found the responses to be reliable and valid, it is possible the data does not capture the full extent of insurer participation in population health networks.

Last, this study measures the extent to which insurers play a role as connectors in community networks, but not the specific nature of those relationships. Further research should consider using mixed methods approaches to better understand the characteristics of insurer engagement in community health and social services networks and the mechanisms by which those relationships might improve capacity and health outcomes.

## IMPLICATIONS

Reducing health inequities between rural Appalachian communities and other geographic areas will require bolstering capacity in both clinical and social services systems. This study suggests that high insurer connectivity in cross-sector community health and social services networks may be an important strategy in decreasing preventable hospitalization rates in rural Appalachia. Policymakers should consider how to craft policies and programs to incentivize insurer engagement in population health efforts. Some evidence suggests that such policy change can spur activities to benefit the wider community. For example, changes to IRS nonprofit hospital regulations may be associated with increased involvement in community benefit activities.^24^ Previous studies have found that insurers participate in health and social services networks at lower rates than most other sectors in a community.^6^,^8^,^19^ Combined these findings, the results of the present study suggest promising opportunities to improve health outcomes and well-being for the people of Appalachia through increased insurer engagement in community networks.

**SUMMARY BOX**

**What is already known about this topic?**

Integrating health and social services in a community may help to both strengthen the local cross-sector network and ensure a patient’s health and social needs are met, thereby reducing the likelihood an individual experiences an adverse health event. Private sector partners, like insurers, may be able to play a critical role in strengthening community networks in areas with less capacity.

**What is added by this report?**

Results from this study suggest substantial geographic variation in the association between greater insurer connectivity in community health / social services networks and the probability of an individual being admitted for a preventable hospitalization. Insurer connectivity in rural Appalachian communities was associated with lower likelihood that an individual was admitted for a preventable hospitalization.

**What are the implications for future research?**

Insurer collaboration in cross-sector community networks may be an important strategy in building capacity and reducing the probability of preventable hospitalization in resource-constrained areas like rural Appalachia.

## Figures and Tables

**Table 1:** Results from logistic regression models estimating the association between insurer connectivity in KY urban, rural non-Appalachian, and rural Appalachian population health networks and the probability of preventable hospitalization, 2018§

	All Regions	Urban	Rural Non-Appalachian	Rural Appalachian
**All preventable hospitalizations**				
Low insurer connectivity	0.003	0.000	0.016	0.025
High insurer connectivity	−0.012	−0.005	−0.019	−0.048*
**Acute hospitalizations**				
Low insurer connectivity	0.001	−0.001	0.001	0.015
High insurer connectivity	−0.004	−0.004	−0.006	−0.018*
**Chronic hospitalizations**				
Low insurer connectivity	0.002	0.002	0.017†	0.011
High insurer connectivity	−0.008	−0.001	−0.014	−0.029*
**Diabetes-related hospitalizations**				
Low insurer connectivity	0.001	0.002	0.003	0.002
High insurer connectivity	0.000	0.003	−0.003	−0.006*
**Observations**	484,850	295,510	57,762	131,578

SOURCE: Authors’ analysis of 2018 NALSYS data linked with patient-level KY hospital discharge data.

NOTES:

* *p* < 0.01

† *p* < 0.05

§ The table shows a marginal effect of insurer connectivity. *Insurer connectivity=0* serves as the reference category. All models also controlled for the following patient and community characteristics: age, sex, race, payer, portion of the population below the poverty level, portion of the population uninsured, population size, geographic location, hospital beds per capita, primary care physicians per 100,000 population, and number of federally qualified health centers in a community. Geographic subgroup models do not include local as a covariate. Standard errors were clustered at the community health and social services network level.

**Table 2:** Results from logistic regression models estimating the association between insurer connectivity in KY rural Appalachian population health networks and the probability of preventable hospitalization based on payer type, 2018¶

	All payers	Medicare	Medicaid	Private insurance
**All preventable hospitalizations**				
Low insurer connectivity	0.025	0.026	0.017	0.037*
High insurer connectivity	−0.048*	−0.063*	−0.034*	−0.016 §
**Acute hospitalizations**				
Low insurer connectivity	0.015	0.020 †	0.003	0.019*
High insurer connectivity	−0.018*	−0.025 †	−0.012*	−0.008*
**Chronic hospitalizations**				
Low insurer connectivity	0.011	0.008	0.015	0.020*
High insurer connectivity	−0.029*	−0.038*	−0.023*	−0.008
**Diabetes-related hospitalizations**				
Low insurer connectivity	0.002	0.000	0.002	0.005*
High insurer connectivity	−0.006*	0.006 †	−0.007	−0.005 †
**Observations**	131,578	70,402	36,420	20,990

SOURCE: Authors’ analysis of 2018 NALSYS data linked with patient-level KY hospital discharge data.

NOTES:

* *p* < 0.01

† *p* < 0.05

§ *p* < 0.10

¶ The table shows a marginal effect of insurer connectivity.

*Insurer connectivity=0* serves as reference category. All models also controlled for the following patient and community characteristics: age, sex, race, payer, portion of the population below the poverty level, portion of the population uninsured, population size, hospital beds per capita, primary care physicians per 100,000 population, and number of federally qualified health centers in a community. Standard errors were clustered at the population health network level. Self-pay/no charge and other insurance type patients were excluded from subgroup analysis because of small sample size.
